# Exploring Electrical Neuromodulation as an Alternative Therapeutic Approach in Inflammatory Bowel Diseases

**DOI:** 10.3390/medicina60050729

**Published:** 2024-04-27

**Authors:** Suofeiya Dilixiati, Jiaxi Yan, De Qingzhuoga, Gengqing Song, Lei Tu

**Affiliations:** 1 Division of Gastroenterology, Union Hospital, Tongji Medical College, Huazhong University of Science and Technology, Wuhan 430022, China; suofeiya@hust.edu.cn (S.D.); dedingzhuoga@hust.edu.cn (D.Q.); 2 Division of Gastroenterology and Hepatology, MetroHealth Medical Center, Case Western Reserve University, Cleveland, OH 44109, USA; jiaxi_yan@ucsb.edu

**Keywords:** inflammatory bowel disease (IBD), ulcerative colitis (UC), Crohn’s Disease (CD), electrical neuromodulation

## Abstract

*Background and Objectives*: This review systematically evaluates the potential of electrical neuromodulation techniques—vagus nerve stimulation (VNS), sacral nerve stimulation (SNS), and tibial nerve stimulation (TNS)—as alternative treatments for inflammatory bowel disease (IBD), including ulcerative colitis (UC) and Crohn’s Disease (CD). It aims to synthesize current evidence on the efficacy and safety of these modalities, addressing the significant burden of IBD on patient quality of life and the limitations of existing pharmacological therapies. *Materials and Methods*: We conducted a comprehensive analysis of studies from PubMed, focusing on research published between 1978 and 2024. The review included animal models and clinical trials investigating the mechanisms, effectiveness, and safety of VNS, SNS, and TNS in IBD management. Special attention was given to the modulation of inflammatory responses and its impact on gastrointestinal motility and functional gastrointestinal disorders associated with IBD. *Results*: Preliminary findings suggest that VNS, SNS, and TNS can significantly reduce inflammatory markers and improve symptoms in IBD patients. These techniques also show potential in treating related gastrointestinal disorders during IBD remission phases. However, the specific mechanisms underlying these benefits remain to be fully elucidated, and there is considerable variability in treatment parameters. *Conclusions*: Electrical neuromodulation holds promise as a novel therapeutic avenue for IBD, offering an alternative to patients who do not respond to traditional treatments or experience adverse effects. The review highlights the need for further rigorous studies to optimize stimulation parameters, understand long-term outcomes, and integrate neuromodulation effectively into IBD treatment protocols.

## 1. Introduction

Inflammatory bowel disease (IBD), including Crohn’s Disease (CD) and ulcerative colitis (UC), represents a spectrum of chronic inflammatory disorders that predominantly disrupt the gastrointestinal (GI) system. Characterized by overlapping clinical features such as diarrhea, abdominal pain, and rectal bleeding, CD and UC differ significantly in their pathophysiological foundations [1]. The genesis of IBD intertwines genetic predispositions with environmental triggers, including shifts in the gut microbiota landscape and enhanced intestinal permeability, orchestrating a dysregulated immune response that ultimately leads to the tissue damage observed in these conditions [2].

Traditionally, the medical management of IBD has been focused on anti-inflammatory therapies, from aminosalicylates to biological agents [3,4]. Although these treatments have been effective for many of the patient population, a significant number either do not achieve adequate symptom control or experience negative side effects, highlighting the imperative for a broader range of treatment options. The increasing incidence of IBD in developing countries introduces further complexities as disparities in access to these advanced therapeutic modalities grow, necessitating the development of effective and accessible care solutions that can be globally implemented [5].

IBD’s relapsing–remitting nature, marked by cycles of flare-ups and quiescence, significantly erodes the quality of life for affected individuals. Despite a plateau in hospitalization rates within developed territories, attributed to therapeutic advancements and enhanced disease surveillance, the escalating global incidence of IBD—potentially fueled by dietary westernization—demands a pivot towards innovative care approaches [6,7,8,9].

**Figure 1:**
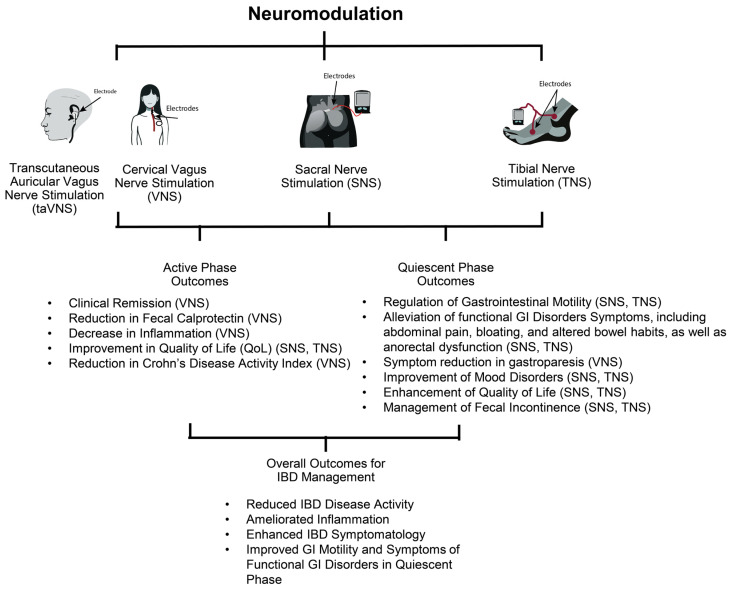
Comprehensive effects of neuromodulation on active and quiescent phases of inflammatory bowel disease (IBD).

Neuromodulation: a frontier in IBD therapy leveraging electrical impulses to recalibrate the nervous system’s regulatory influence over immune activity and GI functionality (Figure 1). In the evolving landscape of neuromodulation, we find a promising avenue for confronting the intricate challenges posed by IBD, integrating symptom relief with potential disease modification strategies. This review examined literature sourced from PubMed, encompassing publications from 1978 through 2024, embarking on an in-depth examination of the current state of neuromodulation in the context of IBD therapy in both animal models and clinical studies. It aims to highlight its potential as a therapeutic tool, clarify the mechanisms by which it exerts its effects, and outline future directions for research in this dynamic field.

## 2. Fundamental Pathophysiological Mechanisms for the Anti-Inflammatory Property of Electrical Neuromodulation

The GI tract engages in a bidirectional dialogue with the central nervous system (CNS), primarily mediated by the vagus nerve (VN), thoracolumbar connections, and the sacral nerve (SN), thus presenting an optimal target for bioelectric neuromodulation therapy [10]. The enteric nervous system (ENS) consists of a vast ensemble of neurons that exert regulatory control over gut immune cells. This ensemble includes intrinsic primary afferent neurons, vasoactive intestinal peptide-secreting neurons, and cholinergic neurons, which are pivotal in the modulation of inflammation-associated signals within the GI milieu [11].

Embedded within the gut–brain axis, the ENS contributes to a functionally interwoven network that encompasses the GI tract, CNS, and the peripheral nervous system (PNS). This network is instrumental in monitoring and modulating the functionality of the GI system. ENS dysfunctions are implicated in the etiology of various functional GI and metabolic disorders, characterized by symptoms such as dysmotility, visceral hypersensitivity, compromised mucosal integrity and immune function, dysbiosis, and altered CNS processing [12].

The VN’s bidirectional pathways—efferent, innervating the ENS and gut immune cells, and afferent, signaling to the CNS—are integral to the systemic anti-inflammatory response. Such pathways are fundamental to the mechanisms by which electrical neuromodulation exerts anti-inflammatory effects within the GI domain [11].

IBD pathogenesis is significantly influenced by the intricate interactions within the gut–brain axis. Disruptions or dysregulations in this nexus of neural networks, along with psycho–neuro–endocrine–immune factors, may precipitate or exacerbate the disease process [13,14]. Functional magnetic resonance imaging studies have revealed distinct patterns of resting-state brain activity in IBD patients, providing insights into the potential correlation between CNS structures, activities, and GI disease manifestations [15].

Notably, the autonomy of the ENS allows for the regulation of key GI functions independently from CNS oversight. This includes the orchestration of peristalsis throughout the intestinal tract, local blood flow regulation, and fluid transmucosal movement. Disorders within the ENS can significantly alter GI motility, leading to complications that intersect with the symptomatology of IBD [16]. Additionally, the ENS plays a role in regulating the proliferation of intestinal epithelial cells and in maintaining the integrity of the intestinal barrier, further influencing the pathophysiological landscape of IBD [17].

The detailed exploration of these neuroimmune interactions and their implications within IBD pathophysiology provides a compelling rationale for the utilization of electrical neuromodulation as a therapeutic strategy, necessitating continued research to fully unravel the nuances of this promising treatment modality.

### 2.1. Central Nervous System Involvement in the Pathophysiology of IBD

The CNS is implicated in the IBD pathophysiology through intricate mechanisms of systemic inflammation originating from the gut [18]. Chronic intestinal inflammation is known to compromise the intestinal barrier, leading to dysbiosis and microbial translocation that, in turn, trigger immune responses with reciprocal CNS effects [19]. This dynamic interplay underscores the relevance of the CNS in the etiopathogenesis of IBD.

The CNS modulates GI function via a network of bidirectional pathways. The vagal afferents relay sensory information from the enteric milieu to the CNS, while the efferents allow CNS-directed modulation of gut motility and secretion. The sympathetic thoracolumbar outflow and sacral nerve innervation are pivotal in their respective excitatory and inhibitory effects on GI activity [20].

Neuroimaging studies have highlighted neurokinin-1 receptor binding potential deficits in IBD patients, akin to those observed in chronic pain conditions, suggesting a neural correlate for the nociceptive and emotional dimensions of IBD [21]. Furthermore, alterations in cortical thickness and neuroplastic changes have been observed, indicative of the CNS’s adaptive response to chronic gut inflammation [22]. The cingulate and insular cortex, areas associated with pain processing, show changes correlating with abdominal pain and depression, prevalent among IBD patients [23,24,25].

These neural processing changes, especially in pain perception, suggest that sensory pathway alterations might contribute to IBD pathophysiology. Dysregulation in brain activity could potentially influence the hypothalamic-pituitary-adrenal (HPA) axis and the ANS, impacting pro-inflammatory mediator production and homeostasis [13]. Research links psychological stress with IBD symptom exacerbation, emphasizing the neuro-immunological connections mediated by the CNS through neurotransmitter and hormonal pathways [26,27,28].

The high prevalence of anxiety disorder has been linked to IBD flares but can persist during remission periods [29]. A combined multidisciplinary approach involving GI physicians and psychologists for anxiety treatment as an adjuvant therapy might benefit disease management [30]. Preliminary studies show promising results of using antidepressants, a subtype of central neuromodulators, as a treatment for IBD in the context of gut–brain dysregulation. Antidepressant therapy may improve sleep and reduce pain, manage chronic diarrhea during IBD remission, and potentially reduce inflammation, thereby reducing disease activity, highlighting the interconnected nature between the neural and GI systems [31].

### 2.2. Enteric Nervous System Involvement in the Pathophysiology of IBD

The ENS is a complex, often described as the “second brain”, a sophisticated network of neurons and glial cells embedded within the GI tract wall. It autonomously orchestrates a wide range of GI functions, including motor activity, local blood flow, and mucosal transport, thereby ensuring the proper functioning of the digestive system [20,32]. This intricate system operates independently but maintains a dynamic, bidirectional communication with CNS, thereby playing a pivotal role in the gut–brain axis—a fundamental concept in understanding IBD pathogenesis.

Recent research underscores the ENS’s susceptibility to chronic psychological stress and systemic inflammatory responses, which can exacerbate IBD symptoms through mechanisms involving neurogenic inflammation and altered neural plasticity [33]. These findings indicate that stress-related neuroendocrine responses can lead to a pro-inflammatory state in the GI tract, further destabilizing gut homeostasis and contributing to the progression of IBD.

The ENS shares many structural and neurochemical characteristics with the CNS, thus their similarity in their susceptibility to certain pathophysiological processes. Diseases typically associated with CNS dysfunction, such as autism spectrum disorder and Parkinson’s disease, often manifest significant enteric symptoms, highlighting the interconnected nature of ENS and CNS [34]. Significant neurochemical remodeling in the myenteric neurons, marked by a shift from cholinergic to peptidergic innervation, was observed in UC patients, suggesting a systemic alteration in the neurochemical coding rather than a localized response to inflammation [35]. Akin to astrocytes in the CNS, enteric glial cells (EGCs) are part of the ENS, and they are integral in maintaining GI track integrity and modulating neuronal activities. EGCs are actively involved in inflammatory and immune processes within the ENS, suggesting a link between EGC dysfunction and the IBD pathophysiology [36]. Another study highlights the therapeutic potential of targeting the ENS in managing IBD. It shows that modulating ENS components, particularly EGCs, can influence immune cell activation and infiltration, reduce inflammation, and reduce the production of ENS-derived pro-inflammatory neuropeptides [37].

The exploration of the ENS involvement in IBD pathophysiology, coupled with emerging evidence on the efficacy of neuromodulatory interventions, underscores the ENS’s significance as a pivotal therapeutic target. This focus not only enhances our understanding of IBD’s complex mechanisms but also positions neuromodulation as a promising strategy for advancing IBD treatment.

### 2.3. Autonomic Nervous System Involvement in the Pathophysiology of IBD 

The ANS is a part of the peripheral nervous system (PNS) that regulates involuntary physiological processes. The ANS includes sympathetic and parasympathetic nervous systems, containing afferent and efferent nerve fibers that relay information from the CNS, bridging the CNS and the body’s involuntary functions [38]. The ANS is responsible for maintaining hemostasis, including regulating the cardiovascular system, GI motility and secretion, and other visceral activities [39,40]. The sympathetic branch has been associated with primary and secondary lymphoid organs, establishing a direct pathway for the brain to influence immune activity. By modulating clonal expansion, cytokine production, and receptor expression, the sympathetic nervous system can either enhance or inhibit the immune response, indicating its role in the overall inflammatory response [41]. The ANS can influence both innate and adaptive immunity. Dysfunctions in the ANS, characterized by imbalances in sympathetic and parasympathetic activities, contribute to the pathology of chronic inflammation and autoimmune conditions [42]. In IBD patients, the ANS, particularly the ENS, undergoes substantial alternations, including changes in neuronal signaling, morphology, and neurotransmitter levels. Those alternations impact intestinal function, contributing to IBD’s pathogenesis [43]. Disorders of the ANS, such as autonomic dysfunction, can result in multisystemic manifestation, including widespread dysmotility throughout the GI tract [44]. The high prevalence of clinically manifested autonomic dysfunction within the IBD population indicates a significant intersection between ANS and IBD. Further, this ANS dysregulation, manifesting as prolonged small bowel transit times, established a direct connection between ANS dysfunction and GI motility symptoms in IBD [45]. Even during clinical remission, IBD patients exhibit significant ANS dysfunction. Notably, UC patients showed reduced parasympathetic activity compared to CD patients and controls, suggesting ANS dysfunction’s role in the pathogenesis of IBD [46].

### 2.4. Vagus Nerve System Involvement in the Pathophysiology of IBD

The VN is integral to the parasympathetic branch of ANS, influencing the neuro-immune axis and demonstrating anti-inflammatory effects in IBS through afferent and efferent pathways. VNS has been shown to reduce IBD activity by attenuating systemic inflammation [47,48]. The VN’s anti-inflammatory mechanisms include the Cholinergic Anti-Inflammatory Pathway (CAIP), where acetylcholine release modulates the immune system. VN’s efferent fibers stimulate glucocorticoid release via the HPA axis, decreasing inflammation. Additionally, the VN also suppresses pro-inflammatory cytokines in the spleen and mesenteric lymph nodes [49,50,51,52].

The VN regulates the HPA axis through vagal afferent pathways that respond to peripheral pro-inflammatory factors like IL-1β and TNF-α, activating the HPA axis. This leads to a cascade involving the release of corticotrophin-releasing factors and adrenocorticotropic hormones, culminating in the release of cortisol, which suppresses inflammation [53,54,55,56]. Thus, the VN’s interaction with the HPA axis is crucial in coordinating the inflammation response, underlining its therapeutic potential in chronic inflammatory diseases like IBD.

CAIP is critical for systemic inflammatory response regulation and functions through acetylcholine released by the VN. The released acetylcholine then acts on macrophages, particularly through the α7 nicotinic acetylcholine receptor (α7 nAChR), reducing pro-inflammatory cytokine production, such as TNFs, IL-1b, IL-6, and IL-18. Notably, VN efferent fibers reduce pro-inflammatory cytokines without boosting anti-inflammatory factors [57,58,59,60]. Nicotine, a cholinergic agonist, shows variable effects on intestinal inflammation [61]. VNS directly stimulates the VN’s efferent branch, triggering acetylcholine release to downregulate inflammation and cytokine secretion, contrasting the slower response of the HPA axis due to its complex neuroendocrine mechanisms [62,63,64].

Additionally, the splenic sympathetic anti-inflammatory pathway, part of CAIP, involves the VN stimulating the splenic sympathetic nerve. This leads to acetylcholine release by lymphocytes, inhibiting TNF-α production by spleen macrophages [65,66]. The sympathetic system also regulates intestinal immunity via the superior mesenteric nerve (MSN), reducing colitis severity [49].

Despite the debate over the VN’s direct interaction with sympathetic innervation in the spleen, recent studies indicate a complex interaction network, including the superior mesenteric ganglion (SMG), in anti-inflammatory processes [67].

## 3. Vagus Nerve Stimulation in IBD Therapy

Decreased parasympathetic activity and reduction in vagal tone were observed in CD patients compared to healthy individuals, with a noted correlation between reduced vagal tone and extended duration of the disease [68]. In patients with newly diagnosed UC, higher parasympathetic activity after the remission of the initial flare was associated with decreased systemic inflammation [69]. These findings provide a robust theoretical basis for employing VNS as a therapeutic strategy aimed at augmenting vagal tone, thereby potentially easing the symptomatic burden of IBD. Recent research has increasingly investigated the effect and therapeutic potential of VNS in managing IBD, highlighting its ability to modulate immune responses and gastrointestinal functionality, potentially offering significant relief from IBD symptoms.

### 3.1. Devices and Mechanisms of VNS

VNS devices have evolved significantly in recent years, with applications now extending beyond refractory epilepsy and depression. Traditional VNS devices require surgical implantation near the neck, linking to a chest-placed pulse generator. Those devices, although increasingly sophisticated, have surgery-related risks. Noninvasive VNS options, like transcutaneous stimulation at the ear or neck, are emerging, offering potential benefits without the risks associated with surgery [70]. Transcutaneous cervical vagal nerve stimulation (tcVNS) was used to treat gastroparesis through self-administered neck stimulation, demonstrating the diversity of VNS applications. However, variations in patient compliance and inconclusive efficacy findings necessitate further research [71,72]. Transcutaneous auricular vagus nerve stimulators (taVNS) target specific ear regions, with a preference for the left ear to avoid potential heart rate effects associated with stimulating the right ear’s auricular branch of the VN [73]. While VNS can induce side effects like hoarseness and throat pain, these can be managed by adjusting stimulation settings. The precise mechanisms underlying VNS therapeutic effects are still being unraveled, but it is hypothesized that VNS may enhance neural activity and blood flow in certain brain areas [74].

VNS parameters, such as stimulation intensity, frequency, pulse width, and pulse number, significantly influence treatment outcomes. Research indicates lower intensity (400 μA) and fewer stimulations (50 pulses) can effectively drive plasticity. Additionally, the interactions between current amplitude and pulse width are crucial, having a more significant impact than changes in pulse number alone. A lower frequency is supposed to show anti-inflammatory potential through efferent branches of VN and CAIP. This supports the use of lower-frequency VNS (1–10 Hz) in treatments like IBD due to its potential anti-inflammatory effects [75,76,77].

### 3.2. VNS Efficacy in Animal Models

The exploration of VNS as a potential treatment for IBD has been extensively investigated in animal models. These studies employ various models, including 2,4,6-trinitrobenzene sulfonic acid (TNBS)-induced colitis, to replicate key IBD symptoms such as bloody diarrhea, weight loss, and mucosal inflammation. These models are crucial for exploring IBD pathogenesis and assessing the efficacy of novel treatments [78,79]. In the VNS experiments, rats underwent surgery to place electrodes around the left cervical vagus nerve. Stimulation parameters, including amplitude (0.25 mA to 3 mA) and frequency (5 Hz to 40 Hz), varied across studies to optimize outcomes. Following VNS, colitis severity was evaluated through clinical signs such as weight loss and changes in stool consistency, alongside direct inflammation measures, including colonic damage assessments, histological examinations, and biochemical analyses for inflammatory markers. Additionally, heart rate variability (HRV) analysis was frequently employed to gauge the ANS response to VNS.

A few animal studies have investigated the therapeutic potential of VNS in a colitis model, specifically in TNBS-induced colitis rat models (Table 1). VNS significantly impacts the modulation of cytokine mRNA levels and substantially affects the multivariate index of colitis. Those findings suggest a more pronounced beneficial effect of VNS on less damaged tissues, indicating its potential utility in preventing the progression of lesions in cases of mild colitis [80]. Furthermore, one of the studies shows VNS’s role in the activation of mitogen-activated protein kinase (MAPK) and the nuclear translocation of NF-κB, both critical pathways in the inflammatory process [81]. Interestingly, another study revealed that while VNS and electroacupuncture independently reduce inflammation, their combination does not significantly improve these anti-inflammatory effects beyond a single treatment [82]. All these studies consistently demonstrated that VNS significantly reduced inflammatory responses in TNBS-induced colitis, evidenced by decreased levels of pro-inflammatory cytokines, improved histological scores, and reduced colitis symptoms. They also noted a significant influence of VNS on autonomic functions, primarily by increasing vagal activity and decreasing sympathetic activity, correlating with reduced inflammation. Collectively, these findings affirm the potential of VNS as a therapeutic strategy in IBD treatment [80,81,82]. While these animal studies provide valuable insights, translating their findings to human IBD treatment involves complexities due to differences in human physiology and IBD pathophysiology. Optimizing VNS parameters for humans remains a challenge, as rodent model parameters do not directly apply due to physiological differences. Furthermore, individual variability in response to VNS in humans highlights the need for careful consideration before applying these findings to clinical settings.

### 3.3. VNS Clinical Trials in IBD

VNS, aimed at modulating the inflammatory reflex pathway, has demonstrated encouraging results in alleviating patients with IBD. A review of the clinical trial landscape reveals a growing body of evidence supporting VNS’s efficacy, with six identified studies—five focusing on CD and one encompassing both CD and UC patients (Table 2). The initial case study in 2014 marked a significant milestone, reporting substantial clinical improvements and EEG alterations. This was followed by a series of pilot studies conducted over durations ranging from 6 to 12 months, consistently indicating reductions in disease activity and inflammation markers. The progression in research culminated in a 2023 multi-center trial spanning 16 weeks, further validating VNS’s role as a viable therapeutic strategy for addressing refractory IBD cases.

Clinical trials of utilizing VNS as an experimental therapeutic treatment for IBD began with a case study of a 49-year-old male with a long-standing history of ileal CD (Crohn’s Disease Activity Index (CDAI) score of 330). After 12 months of VNS (initiated at 0.5 mA, increased to 1 mA, with a frequency of 10 Hz), significant electroencephalogram (EEG) changes were observed across various frequency bands, especially in the theta and gamma bands. Most notably, the patient’s CDAI score decreased to remission levels, and endoscopic remission was achieved. An increase in high-frequency power in heart rate variability (HRV) analysis indicated the enhanced parasympathetic tone. In addition, the patient remained in remission for an extended period (27 months) after the VNS treatment, indicating long-term benefits and the potential for sustained improvement. The study is based on a single patient, which significantly limits the generalizability of the findings. In addition, as the study acknowledges, the possibility of a placebo effect or improvement unrelated to VNS treatment cannot be completely ruled out. The long-term safety and efficacy, particularly in a larger population, remain to be established. Despite those limitations, these promising results open possibilities for further research [86].

Building on these initial findings, subsequent investigations, including an open-label study recruiting seven active CD patients, were conducted. Over six months of VNS therapy, a majority exhibited improvements in their CDAI scores, with a significant number reaching clinical remission. A noteworthy reduction in fecal calprotectin (FC) levels further corroborated these findings. Despite the absence of a control group, raising concerns about potential placebo effects and the inherent bias of open-label designs, these studies underscore the therapeutic promise of VNS in IBD management [87].

Extended observation revealed neurophysiological impacts of VNS, such as acute and chronic shifts in EEG power spectra and modifications in HRV, indicative of reduced stress levels among participants. A subsequent 12-month clinical evaluation demonstrated that a significant proportion of patients with moderate CD achieved clinical remission, further validating VNS’s role in IBD therapy. Despite the absence of a control group and blinding, variability in neurological responses was noted, suggesting differential VNS effects among individuals. While EEG modifications correlate with clinical improvements, elucidating the precise mechanisms by which VNS influences brain activity and symptomatology in IBD remains an area for future investigation [88].

In another study, 71% of patients with moderately active CD achieved clinical remission, with CDEIS scores significantly reduced, indicating both clinical and endoscopic remission. This outcome, alongside decreased CRP and fecal calprotectin levels, underscores VNS’s potential for managing moderate CD. Nevertheless, the exclusion of two patients due to deteriorating conditions and the study’s acknowledgment of VNS’s gradual effect highlight its possible limitations for only mild to moderate CD cases [89].

**Table 2:** Summary of clinical trials on electrical neurostimulation in inflammatory bowel disease (IBD) and other conditions.

Diseases	Population Size	Stimulation Sites	Pulse Features	Stimulation and Treatment Durations	Major Findings	Year	References
CD and UC	23 patients (1 withdraw due to infection); 10 CD, 12 UC	Ta-VNS at Cymba conchae of the external left ear	20 Hz, 0.3 ms pulse width, 300 s continuous ON	5 min once daily (first 2 weeks); 5 min twice daily (Week 4 to 16)	Achieved clinical remission; reduced fecal calprotectin; improved quality of life	2023	[90]
CD	17 patients (16 analyzed)	VNS at left cervical vagus nerve	0.25–2.0 mA, in 0.25 mA increments, 0.25 ms pulse width	1 min once per day, gradually increased to 5 min; 16 weeks	Reduced CDAI; reduced fecal calprotectin; decreased mucosal inflammation; reduced serum levels of inflammatory cytokines; improved quality of life	2023	[91]
CD	9 patients	VNS at left cervical vagus nerve	0.25 mA, 10 Hz, 0.25 or 0.5 ms pulse width, 30 s ON, 300 s OFF	12 months	Achieved clinical and endoscopic remission; decreased C-reactive protein and fecal calprotectin; restored vagal tone and reduced digestive pain; changed cytokine profile	2020	[89]
CD	9 patients	VNS	0.5–1.25 mA, 10 Hz, 0.5 ms pulse width, 30 s ON, 300 s OFF	12 months	Reduced alpha activity was associated with improved clinical outcomes, reduced anxiety, and decreased fecal calprotectin	2018	[88]
CD	7 patients	VNS at left cervical vagus nerve	0.25–1.25 mA, 10 Hz, 0.5 ms pulse width, 30 s ON, 300 s OFF	6 months	Achieved clinical, biological, and endoscopic remission, with restored vagal tone; decreased CDAI, C-reactive protein, and fecal calprotectin	2016	[87]
CD	A 49-year-old male	VNS at left cervical vagus nerve	0.5–1 mA in 0.25 mA increments, 10 Hz, 0.5 ms pulse width, 30 s ON, 300 s OFF	12 months	Achieved endoscopic remission; decreased CDAI, with increased parasympathetic tone	2014	[86]
FI; OAB; UR; BPS/IC; FI; DI	Patients receiving implantable pulse generator: 20 OAB, 21 UR, 12 BPS/IC, 7 FI, 4 DI.	SNS at S3 sacral nerve root	N/A	14–220 months	Improved clinical symptoms, quality of life, and satisfaction	2021	[92]
FI; Low Anterior Resection Syndrome	10 patients	SNS at S3 and S4 of sacral nerve	N/A	14.7 days mean test stimulation	Significantly improved low anterior resection syndrome and fecal incontinence quality of life score; avoided permanent colostomy	2021	[93]
UP in UC Patients	A 58-year-old female	SNS	0.5–1.5 V, 14 Hz, 210 ms pulse width	3-week temporary stimulation; 18 months permanent stimulation	Improved endoscopic and histologic scores; decreased rectal barrier permeability	2015	[94]
FI in CD	5 patients	SNS at S3 sacral nerve	5 Hz, 0.1 ms pulse width	3-week initial stimulation; 3–36 months permanent stimulation	Improved continence, with significant decrease in the Wexner scores; improved quality of life	2008	[95]
CD, UC, and Undetermined Colitis	12 patients (7 CD, 3 UC, 2 Undermined Colitis)	Posterior TNS (PTNS)	10–30 mA, 10Hz, 0.2 ms pulse width	Several minutes per day; 3 months	Improved continence; improved quality of life	2009	[96]

Please note: mixed conditions include overactive bladder (OAB), urinary retention (UR), bladder pain syndrome/interstitial cystitis (BPS/IC), double incontinence (DI), and fecal incontinence (FI).

In a more recent open-label clinical trial involving 17 patients with treatment-refractory CD, VNS demonstrated significant therapeutic benefits. A notable average reduction in CDAI of −86.2 ± 92.8 in the entire cohort and −114.5 ± 23.9 in the stimulation monotherapy group was observed at Week 16. Additionally, there was a significant reduction in fecal calprotectin levels from 5054 ± 1266 to 1969 ± 625.5 μg/g in all patient groups and from 4705 ± 1295 to 1496 ± 579 μg/g in the monotherapy group. Serum cytokines also showed notable reductions, with tumor necrosis factor and interferon-γ decreasing by 46 and 52%, respectively, while mean total IL-17 levels were 54% higher at Week 16 than baseline. Additionally, 27% of all patients (co-treatment with biologics) and 36% of those on VNS mono treatment achieved clinical remission (CDAI < 150) at Week 16. Quality of life improvements were reported in the Inflammatory Bowel Disease Questionnaire (IBDQ) and the Simple Health Score (SHS). Among patients in the monotherapy group, 54% (6 out of 11) exceeded the “minimal important difference” in the IBDQ, and a majority of patients reported improvements in their SHS. The treatment was generally safe and well tolerated, with only one severe adverse event related to a postoperative infection requiring device explanation being reported, representing 5% of the study population. The applicability of the findings to a broader IBD patient population might be limited because the participants included were all refractory to biologic treatments. In addition to the small sample size, lack of blinding, and placebo control, the short duration of the study may overlook the long-term efficacy and safety of VNS [91].

Owing to the risks associated with surgically implanting electrodes on the VN, alternative stimulation methods are highly anticipated, especially for vulnerable populations. A partial blinding study conducted in 2023 evaluated the efficacy and safety of ta-VNS in pediatric patients with CD and UC. Clinical remission was achieved in 50% (3 out of 6) with CD and 33% (2 out of 6) UC patients who had mild to moderate symptoms activity (6 out of 10 CD and 6 out of 12 UC patients). Meanwhile, 64.7% (11 out of 17) of subjects with baseline fecal calprotectin levels over 200 μg/g showed a greater than 50% reduction in fecal calprotectin level at week 16, with UC subjects showing 81% median reduction and CD subjects showing a 51% median reduction comparing to baseline. Reduced anxiety among patients was also reported. ta-VNS treatment was well-tolerated, and no serious adverse events were reported, highlighting its potential suitability for pediatric patients. The study validates that ta-VNS is a low-risk intervention and effective noninvasive treatment for mild to moderate IBD in a pediatric population, showing significant symptom improvement and inflammation reduction, warranting further investigation with larger, placebo-controlled trials in a broader patient population [90].

The limitations of small sample sizes and the variability in disease severity and individual responses limit the generalizability of these studies, highlighting the need for more rigorous, controlled research to firmly establish VNS’s efficacy in IBD treatment.

## 4. Other Methods of Electrical Neuromodulation

As the exploration of electrical neuromodulation expands beyond VNS for IBD treatment, interest has grown in the potential of sacral and tibial nerve stimulation. These methods, while less researched, offer intriguing possibilities for management in IBD patients.

### 4.1. Sacral Nerve Stimulation in IBD Therapy

The sacral nerve (SN), specifically the anterior sacral roots S2, S3, and S4, are pivotal in innervating the sigmoid colon, rectum, and external anal sphincter and in controlling bladder and genital organ functions. Studies reveal that electrical stimulation of these roots affects colorectal motility differently: S2 induces low-pressure contractions, S3 initiates high-pressure activity, and S4 enhances tone and sphincter activity. The pelvic nerves, emerging from these sacral roots, supply the colorectum, impacting bowel and bladder control and sexual functions. SNS, targeting these nerves, is an effective therapy for bladder and bowel dysfunctions unresponsive to conventional treatments [10,48,97]. SNS received FDA approval in 1997 for treating urinary incontinence in the U.S. With further research, this approval was extended for urinary retention and overactive bladder in 1999 and for fecal incontinence in 2011. The FDA’s recognition of SNS for bowel dysfunctions highlights its potential utility in IBD management, underscoring the need for targeted research to explore its efficacy in this area.

### 4.2. SNS Efficacy in Animal Models

Research on SNS in animal models of colitis shows its potential to mitigate colonic inflammation. Studies like Pasricha et al. have found significant reductions in Disease Activity Index (DAI), histological improvements, and a 57% decrease in colonic myeloperoxidase activity, alongside diminished TNF-α expression and an increase in M2 macrophage populations [84]. These findings are complemented by HRV analyses indicating an enhanced vagal tone. Similarly, Tu et al. reported SNS-induced improvements in autonomic function, evidenced by upregulated vagal efferent activity and reduced plasma norepinephrine, correlating with decreased DAI and inflammatory markers in colonic tissues, including a reduction in pro-inflammatory cytokines (TNF-α, IL-2, and IL-17A) and an increase in IL-10 [83].

Exploration of optimal SNS parameters reveals that one hour of daily stimulation offers more pronounced benefits in reducing colonic inflammation than either shorter or longer periods. Bipolar stimulation, which involves placing two electrodes near each other on a single nerve root, significantly outperforms bilateral or unipolar configurations in diminishing inflammation. Lower frequency stimulation at 5 Hz, as opposed to 14 Hz, favorably modulates autonomic activity by enhancing vagal and reducing sympathetic responses. This aligns with findings of decreased pro-inflammatory cytokines (e.g., IL-2, IL-13) and increased anti-inflammatory markers (e.g., IL-10), underscoring the therapeutic potential of specific SNS techniques [85].

However, these insights stem from rodent studies, which inherently limit their applicability to human IBD due to differences in physiology and the disease’s complexity. Short-term experimental designs may not adequately reflect long-term safety and therapeutic outcomes necessary for clinical application.

### 4.3. SNS Clinical Studies in IBD and Other Conditions

The SNS clinical trials, originally intended to address conditions like overactive bladder (OAB), urinary retention (UR), bladder pain syndrome/interstitial cystitis (BPS/IC), fecal incontinence (FI), and diurnal incontinence (DI), have shown promising results. Four out of five of these trials, while not initially targeting IBD treatment, have demonstrated secondary outcomes that suggest a potential role for SNS in effectively managing IBD.

SNS has demonstrated varying degrees of effectiveness in treating bowel-related diseases across different studies. In 2008, an observational study focusing on five CD patients with fecal incontinence found that SNS led to improved continence, with notable reductions in Wexner’s score from 15 to 6 and daily stool frequency from 7 to 2 times, highlighting SNS’s effects in this subset of CD patients over a median of 14 months (range 3–36). However, this study comes with its limitations, including a small sample size of only five patients, hindering the generalizability of its results. The lack of a control group and relatively short follow-up period for some patients challenge long-term efficacy and safety assessment. All participants selected had severe symptoms, resulting in limited applicability to those with less severe manifestations [95].

A recent case study on a patient with refractory ulcerative proctitis (UP) undergoing sacral nerve stimulation (SNS) demonstrated notable improvements in fecal incontinence, disease activity, and histological outcomes. Notably, the Lichtiger Score, which assesses disease activity, dropped from 11 to 4, a reduction maintained through the 18-month follow-up. Fecal incontinence, measured by the Wexner Score, improved significantly, with a slight increase in stability observed over time. Endoscopic and histological assessments, indicated by the Mayo and Geboes Scores, showed sustained improvements. This case also highlighted a reduction in daily fecal leaks and improved rectal barrier function, underscoring SNS’s potential beyond traditional incontinence treatment. Despite these promising results, the study’s observational nature and the lack of a control group limit the generalizability of findings, suggesting the need for more rigorous research [94].

A retrospective analysis involving 10 patients with severe defecation issues post-intersphincteric resection revealed SNS achieved a 40% positive response rate. Notably, median daily bowel frequency reduced from 10 to 6.5, and weekly fecal incontinence episodes decreased from 7 to 4. Although changes in the Wexner score were not statistically significant, 70% of participants avoided the necessity for a permanent colostomy after SNS therapy, indicating its potential to significantly impact surgical outcomes. However, this study’s retrospective nature, small cohort, and lack of a control group limit the extrapolation of these results [93].

In a broader longitudinal study of 106 individuals with diverse pelvic floor disorders, SNS demonstrated substantial benefits over the long term. Among fecal incontinence (FI) sufferers, over 71% reported symptom improvement, with about 14% achieving complete resolution. Yet, one-third of patients witnessed a reduction in therapeutic effects after 75 months, pointing to potential long-term efficacy issues. Device-related discomfort was reported by 39% of patients, though the majority found relief through device adjustment; a small fraction underwent surgical intervention or device removal. Despite these challenges, SNS remains a minimally invasive, effective, and safe option, enhancing symptom management and life quality for many, underscoring its potential utility in IBD management within specific patient subsets [92].

These findings underscore the need for rigorous, controlled studies to better understand SNS’s role in IBD therapy and its long-term implications.

### 4.4. Tibial Nerve Stimulation in IBD Therapy and Other Conditions

Research in this field is still in its early stages. With limited but promising studies on transcutaneous posterior tibial nerve stimulation (TPTNS) and percutaneous posterior tibial nerve stimulation (PTNS), this novel approach suggests a potential to alleviate symptoms for patients experiencing fecal incontinence and other bowel dysfunctions associated with IBD.

One study reviewed the efficacy of TPTNS as a treatment for fecal incontinence in IBD patients. A cohort of 12 patients with different durations of IBD was included. Electrical stimulation to the posterior tibial nerve was delivered by self-adhesive electrodes positioned behind the internal malleolus. After the 3-month treatment period, 41.6% of the patients (5 out of 12) reported significant improvements in symptoms and quality of life. However, only 8% of the patients (1 out of 12) exhibited a significant change in the Wexner score. Among the patients who reported improvements, quality of life enhancements were noted to be over 50%. Despite these positive findings, the improvement in Wexner scores was not generalizable among the participants, leading to consideration of the variability in response to TENS therapy. Additionally, the small sample size and the absence of a control group undermines the ability to establish concrete evidence of TENS’s efficacy, as factors such as natural disease progression or placebo effects cannot be ruled out. The lack of standardization in concomitant treatments and the absence of objective physiological measurements further limit the study’s conclusiveness, underscoring the need for more comprehensive research in this area [96]. TPTNS is also studied in treating functional non-retentive fecal incontinence in children. The results showed a significant decrease in the incontinence score with TPTNS treatment compared to dietetic regulation and Kegel exercises, but less effective compared to biofeedback [98].

PTNS is another form of TNS where a needle electrode is inserted above the medial malleolus and slightly posterior to the tibia. In patients with multiple sclerosis, PTNS showed positive results in treating neurogenic bowel dysfunctions, specifically FI and functional constipation [99]. The treatment led to a significant reduction in the median Cleveland Clinic Fecal Incontinence Score of 29% (12.0 to 8.5), particularly improving incontinence for liquid, flatal incontinence, pads’ need, and lifestyle restrictions, indicating that PTNS is an effective and minimally invasive treatment for MS patients suffering from neurogenic bowel dysfunctions. However, this result should be cautiously interpreted. The study’s limited sample size restricted its ability to effectively identify clinical and demographic predictors of a positive response to PTNS. Furthermore, the pathophysiological mechanism underlying bowel dysfunction may differ significantly between MS and IBD. Therefore, the findings of this study might not be directly applicable or generalizable to patients with IBD [99]. Other studies have also shown that PTNS can improve symptoms and quality of life for patients with combined medical treatment in different GI disorders, highlighting the potential of TNS as an adjunctive treatment in managing IBD-related symptoms [100,101].

In summary, TNS shows promise for symptomatic relief in IBD, but current evidence is limited by small studies, the absence of control groups, and lack of long-term outcomes. Given the distinct pathophysiological differences between IBD and other conditions like multiple sclerosis, careful consideration of these initial findings is necessary. Further, robust research is needed to confirm TNS’s effectiveness as an adjunctive IBD treatment.

## 5. Electrical Neuromodulation for Concurrent GI Motility Disorders in Quiescent IBD

Given the substantial overlap between symptoms of GI motility disorders and functional GI disorders (FGID) in quiescent IBD patients [102], including irritable bowel syndrome (IBS) [103], anorectal dysfunction [104], and mood disorders [105], neuromodulation emerges as a promising strategy for managing these conditions. The intertwining of IBS and IBD, through shared genetic, immunological underpinnings and alterations in the microbiota, underscores the necessity of a nuanced approach to treating the complex symptomatology observed in IBD patients during remission phases [106,107].

SNS is extensively applied in the treatment of fecal incontinence, with proven efficacy, as discussed earlier in this review [92,93,95]. Beyond its established role, SNS has also emerged as a viable option for managing bladder dysfunctions, notably demonstrating significant symptom alleviation in overactive bladder cases among adults [108]. Furthermore, SNS has shown benefits in ameliorating various urinary symptoms, particularly for patients facing refractory urinary urgency–frequency issues [109]. Moreover, different forms of electrical stimulation have shown promise in addressing a range of GI disorders, indicating the versatile potential of neuromodulation in this domain.

The occurrence of IBS-like symptoms in IBD patients in remission not only diminishes their quality of life but is also frequently associated with heightened levels of anxiety and depression. Remarkably, a significant fraction of IBD patients in remission report symptoms meeting the diagnostic criteria for IBS, with a higher prevalence observed in CD than in UC. This correlation underscores the intricate relationship between IBS-type symptoms and psychological well-being in IBD patients, emphasizing the importance of integrating neuromodulation into comprehensive care strategies to address both the physical and emotional facets of these conditions effectively [110].

Experimental models and clinical trials reveal the potential of SNS for treating visceral hypersensitivity in IBS, a common comorbidity in quiescent IBD. In rat models, SNS markedly reduced visceral hypersensitivity, indicated by lower abdominal withdrawal reflex scores and improved autonomic functions [111]. Similarly, a randomized, double-blind, placebo-controlled crossover study observed a marginal but notable reduction in IBS symptoms with sub-sensory SNS in patients with diarrhea-predominant or mixed IBS. Due to its small sample size and short duration, it limits the statistical power and generalizability and may overlook the long-term efficacy and safety. In addition, a notable placebo effect (52% of patients) was observed, hindering the assessment of the true therapeutic effect of SNS [112].

Additionally, spinal cord stimulation, applied at the T5–T8 spinal level, demonstrated potential in reducing pain intensity and frequency of diarrhea episodes in a pilot study despite the absence of statistical significance. The choice of a majority of participants to retain their devices post-trial suggests a subjective perceived benefit. Yet, the small cohort size and methodological constraints limit the generalizability of these results and the establishment of a definitive causal link between neuromodulation and symptom relief [113]. These preliminary findings underscore the necessity for further, more expansive research to thoroughly evaluate the utility of neuromodulation in managing IBS symptoms within the IBD population during remission phases, ensuring a holistic understanding of its efficacy and safety profiles.

In summary, electrical neuromodulation offers a promising approach to managing GI motility and functional disorders in quiescent IBD, particularly for symptoms similar to IBS, anorectal dysfunction, and mood disturbances. SNS exemplifies this potential, especially in enhancing life quality for patients in remission by addressing both physical symptoms and emotional well-being.

## 6. Conclusions

In conclusion, the intricate gut–brain nexus underscores the pathogenesis of IBD, with electrical neurostimulation emerging as a promising avenue for treatment. The potential of VNS to modulate inflammatory responses through the HPA and cholinergic anti-inflammatory pathways has been highlighted in both preclinical and clinical settings. Despite promising outcomes in managing IBD and its associated symptoms like FI and IBS, the need for further research to ascertain the long-term efficacy and safety of VNS remains. Notably, the specific mechanisms underlying the benefits of neuromodulations are not fully elucidated, and there is considerable variability in treatment parameters such as stimulation intensity, frequency, and duration across studies, complicating the comparison of results and the formulation of standardized protocols.

SNS and TNS, though less explored, offer alternative neuromodulatory approaches, especially for IBD patients experiencing concurrent GI motility and functional GI disorders. Preliminary evidence suggests that these modalities may effectively manage specific symptoms, underscoring the potential for broader therapeutic applications.

However, the current landscape of electrical neuromodulation in IBD management primarily focuses on symptom alleviation, with a significant gap in understanding its effects on disease trajectory and progression. The advent of bioelectrical modulation holds promise, yet the minimally invasive nature of VNS, SNS, or TNS does not preclude them from potential side effects, necessitating personalized parameter adjustments.

The heterogeneity in IBD highlights the imperative for tailored treatments, advocating for a comprehensive investigation into neurostimulation parameters to optimize therapeutic strategies. Additionally, while our review outlines promising preliminary findings, we acknowledge the need for more comprehensive studies to confirm these results and understand the long-term implications of neuromodulation in IBD. Despite the absence of a consensus on the synergistic potential with conventional therapies, the evolving evidence base signals a need for further refinement and research in bioelectrical modulation to establish its place within the broader IBD treatment paradigm.

## Figures and Tables

**Table 1:** Summary of electrical neuromodulation studies in animal models.

Animal Models	Disease Models	Stimulation Type	Electrodes Locations	Pulse Features	Stimulation and Treatment Durations	Major Findings	References
Adult male Sprague-Dawley rats	TNBS-induced colitis	VNS	Wrapped around the left cervical vagus nerve and carotid	1 mA, 5Hz, 500 us interval, 10 s ON, 90 s OFF; continuous cycle	3 h per day; 5 consecutive days	Reduced weight loss, improved histology, lessened colitis and inflammation	[80]
Adult male and female Sprague-Dawley rats	TNBS-induced colitis	VNS	Bipolar coil electrodes were placed around the left cervical vagus nerve and left carotid artery	0.25 mA, 20 Hz, 500 ms pulse width, 30 s ON, 5 min OFF continuously	3 h per day; 6 consecutive days	Lowered disease activity, reduced colonic damage, decreased myeloperoxidase, NO synthase, TNF-α, IL-6; inhibited MAPKs phosphorylation and NF-kB p65 translocation	[81]
Adult male Sprague-Dawley rats	TNBS-induced colitis	VNS	One pair of electrodes implanted around the left cervical vagal nerve, 3–5 mm apart	VNS1: 1.0–3.0 mA, 25 Hz, 0.5 ms pulse width, 2 s ON, 3 s OFF VNS2: 1.0–3.0 mA. 40 Hz, 0.5 ms pulse width, 2 s ON, 3 s OFF VNS3: 1.0–3.0 mA, 5 Hz, 0.5 ms pulse width, 10 s ON, 90 s OFF	3 h daily; 21 consecutive days	Reduced DAI, improved macroscopic and histological scores, decreased pro-inflammatory cytokines, increased vagal and decreased sympathetic activity	[82]
Adult male Sprague-Dawley rats	TNBS-induced colitis	SNS	One pair of electrodes placed around the S3 nerve behind the sacral foramen	N/A mA, 5 Hz, 0.5 ms pulse width, 10 s ON, 90 s OFF	1 h daily; 10 consecutive days	Lowered DAI, normalized colon length, increased acetylcholine and anti-inflammatory cytokines, decreased pro-inflammatory cytokines	[83]
Adult male Sprague-Dawley rats	Colitis induced by 5% DSS	SNS	One pair of electrodes placed around S3 right sacral nerve	N/A mA, 5 Hz, 0.5 ms pulse width, 10 s ON, 90 s OFF	1 h daily; 10 consecutive days	Reduced DAI and colonic damage, improved histology, lowered TNF-α, altered neurotransmitter levels	[84]
Adult male Sprague-Dawley rats	TNBS-induced colitis	SNS	Bipolar: one pair of electrodes placed circumferentially around the nerve behind the right S3 sacral nerve, 3–4 mm apart; Unipolar: one electrode placed circumferentially around the nerve behind the right S3 sacral root; second electrode sutured on the muscle 10 mm apart; Bilateral: two electrodes in pair placed circumferentially around the right and left sacral roots	Optimized: N/A mA, 5 Hz, 0.5 ms pulse width, 10 s ON, 90 s OFF; Alternative: N/A mA, 14 Hz, 0.21 ms pulse width, continuous stimulation	0.5 h, 1 h, and 3 h daily, with 1 h being the most effective; 10 consecutive days	1h daily, 5 Hz bipolar SNS most effective; lowered DAI, reduced inflammation, balanced cytokine levels	[85]

Please note: trinitrobenzene sulfonic acid (TNBS).
